# Evaluation of Storage Tubes for Combined Analysis of Circulating Nucleic Acids in Liquid Biopsies

**DOI:** 10.3390/ijms20030704

**Published:** 2019-02-06

**Authors:** Aoife Ward Gahlawat, Judith Lenhardt, Tania Witte, Denise Keitel, Anna Kaufhold, Kendra K Maass, Kristian W Pajtler, Christof Sohn, Sarah Schott

**Affiliations:** 1 Department of Gynecology and Obstetrics, University Hospital of Heidelberg, 69120 Heidelberg, Germany; Aoife.Gahlawat@med.uni-heidelberg.de (A.W.G.); J.Lenhardt@stud.uni-heidelberg.de (J.L.); Tania.WitteTobar@med.uni-heidelberg.de (T.W.); Denise.Keitel@med.uni-heidelberg.de (D.K.); Christof.Sohn@med.uni-heidelberg.de (C.S.); 2 Division of Pediatric Neuro-oncology, German Cancer Research Center (DKFZ), 69120 Heidelberg, Germany; a.kaufhold@kitz-heidelberg.de (A.K.); k.maass@kitz-heidelberg.de (K.K.M.); k.pajtler@kitz-heidelberg.de (K.W.P.); 3 Department of Pediatric Oncology, Hematology and Immunology, University Hospital of Heidelberg, 69120 Heidelberg, Germany

**Keywords:** circulating miRNA, cell-free DNA, blood storage, blood collection tubes

## Abstract

In the last decade, circulating nucleic acids such as microRNAs (miRNAs) and cell-free DNA (cfDNA) have become increasingly important in serving as potential novel biomarkers for a variety of human diseases. If cell-free nucleic acids are to become routinely used in diagnostics, the difference in plasma miRNA and cfDNA levels between healthy and diseased subjects must exceed pre-analytical and analytical variability. Until now, few studies have addressed the time limitations of pre-processing or explored the potential use of long-term blood storage tubes, which might need to be implemented in real-life diagnostics. In this study, we analyzed the stability of four breast cancer-associated miRNAs and two cancer-associated genes under various storage conditions, to test their limitations for potential application in clinical diagnostics. In two consecutive experiments, we tested the limits of conventional EDTA tubes, as well as long-term storage blood collection tubes (BCTs) from four different manufacturers. We found that circulating miRNAs are relatively stable when stored in EDTA monovettes for up to 12 h before processing. When stored in BCTs, circulating miRNAs and cfDNA are stable for up to 7 days, depending on the manufacturer. Norgen tubes were superior for cfDNA yield, while Streck tubes performed the worst in our study with hemolysis induction. In conclusion, plasma prepared from whole blood is suitable for the quantification of both cf-miRNAs and cfDNA simultaneously.

## 1. Introduction

Over the last decade, circulating microRNAs (miRNAs) and cell-free DNA (cfDNA) have increasingly become of interest as novel biomarkers for a variety of human diseases, including cancer [1,2]. Moreover, liquid biopsies have many advantages compared to conventional diagnostic techniques, including their non-invasive nature and the possibility to perform repetitive sampling during the course of disease. 

miRNAs, a group of short noncoding RNAs which regulate gene expression on a posttranscriptional level, are involved in tumorigenesis [3,4,5]. Aside from the cellular form, circulating cell-free miRNAs (cf-miRNAs) have been shown to be remarkably stable in body fluids such as plasma, making them a potential non-invasive diagnostic tool for early cancer detection [6,7,8]. cfDNA found circulating in the blood is proposed to stem, in varying ratios, from DNA released from tumor cells together with DNA fragments from normal cells [9]. With cfDNA, one has the potential to analyze specific alterations coming from the tumor site such as mutations or DNA methylation. Furthermore, combinations of molecular markers in the blood might increase their specificity and sensitivity in diagnostics. Recently, a combination of a panel of DNA mutations and proteins detected in plasma was able to detect five different cancer types with a sensitivity of 69–98% and a specificity of >99% [10]. The combinations of cfDNA and cf-miRNA may be equally powerful as biomarkers in cancer with complementary prognostic values regarding specificity and sensitivity in liquid biopsies. 

However, it remains unclear how long and under what conditions blood can be stored in collection devices before further processing into plasma for cf-miRNA and cfDNA analysis, impacting their potential clinical use. One major pre-analytical challenge is the contamination of circulating nucleic acid with cellular miRNAs and DNA from hematopoetic cells, as a result of in vitro hemolysis. For miRNAs, it has been shown that levels of miR-16 and miR-451a, both of which are found in red blood cells, correlate positively with hemolysis and increase over incubation time [11]. For cfDNA, ruptured blood cells were described to be a main source of cfDNA contamination, being in part avoidable by improved pre-analytical processing [12]. Previous studies have set a strict time limit of processing blood samples within 2 h after collection while storing them at 4 °C [13]. However, in real-life clinical practice, blood drawn for potential diagnostic tests would need to be either stored for up to 24 h or shipped to an external analysis center until further processing, which could take a few days. 

In recent years, long-term blood collection tubes (BCTs) have been developed particularly for this purpose. These tubes are pre-coated with preservatives to prevent cell lysis and, therefore, reduce the release of RNA and DNA from hematopoetic cells [14,15]. While numerous studies have compared BCTs from different manufacturers, until now there has been no consensus as to which device is the best and, in particular, which device is best suited for multi-marker studies such as the combination of cf-miRNA and cfDNA intended for eventual clinical use. The selection of an optimal BCT is essential in the successful clinical establishment of liquid biopsy analysis. In the current study, we evaluated and compared the utility of long-term BCTs from four different vendors in preserving circulating nucleic acids in blood sampled for routine molecular diagnostics.

## 2. Results

### 2.1. Circulating miRNAs Are Stable in EDTA Monovettes for up to 12 h

**Figure 1:**
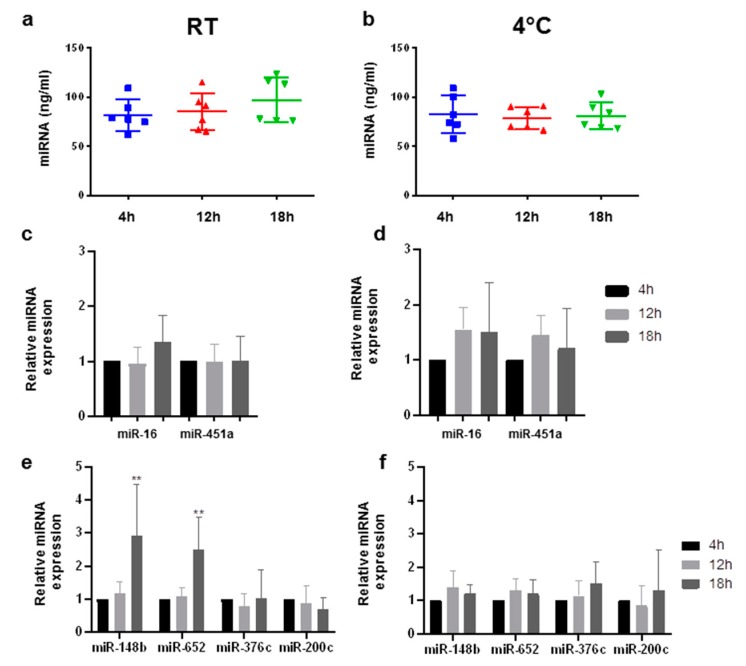
Stability of cell-free microRNAs (cf-miRNAs) stored in EDTA monovettes. Content of cf-miRNA (ng/mL) in plasma after storing blood for 4, 12, and 18 h at room temperature (RT) (**a**) and 4 °C (**b**) in six healthy subjects. Bar plots showing enrichment of miR-16 and miR-451a after 12 and 18 h, relative to 4 h, stored at RT (**c**) and 4 °C (**d**). qRT-PCR results showing the differences in miR-148b, miR-652, miR-376c, and miR-200c after 12 and 18 h stored at RT (**e**) and 4 °C (**f**), relative to 4 h. Stars represent level of significance with (**) for *p*-value < 0.01.

In order to determine the stability of cf-miRNAs after storage in EDTA monovettes, blood was drawn from six healthy individuals and stored at either room temperature (RT) or 4 °C for up to 18 h. Total cf-miRNA slightly increased in three out of six of the samples which were stored at RT (Figure 1a), while no differences were observed in plasma stored at 4 °C for up to 18 h (Figure 1b). Next, we analyzed miRNAsknown to be highly expressed in blood cells, namely miR-16 and miR-451a, as these are established hemolysis markers [11,16]. Storage at RT for up to 18 h did not have any effect on miR-451a levels, whereas samples stored at 4 °C before processing had slightly elevated levels of miR-451a after 12 and 18 h, with corresponding elevations observed for miR-16 (Figure 1c,d). As our aim was to test the limits of blood storage tubes to be implemented in clinical diagnostics, we next analyzed a panel of breast cancer-associated miRNAs, namely miR-148b, -652, -376c, and -200c [13,17,18]. In samples stored at RT, there was a significant increase in miR-148b and -652 in three out of six samples after 18 h (Figure 1e) which was not observed in samples stored at 4 °C (Figure 1f). miR-376c and miR-200c were relatively stable for up to 18 h at both temperatures (Figure 1e,f). A strong correlation was observed among all miRNAs tested in EDTA tubes (Supplemental Figure S1).

### 2.2. Circulating miRNAs Are Stable in Long-Term Storage Blood Collecting Tubes for up to 1 Week

**Figure 2:**
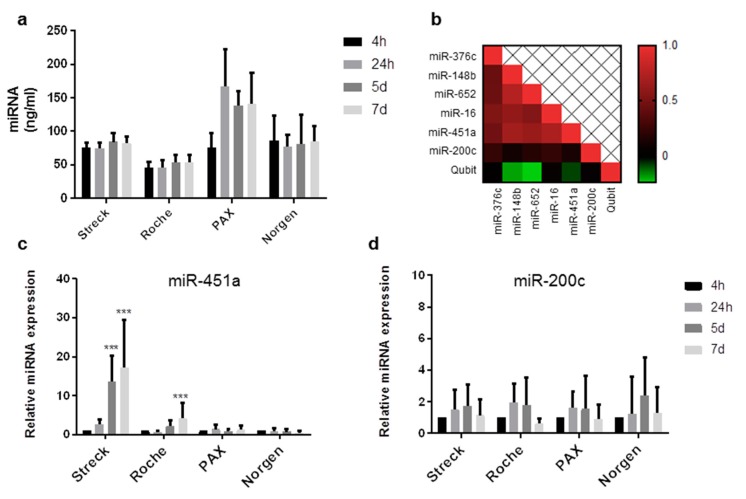
Stability of cf-miRNAs stored in long-term storage tubes. Content of cf-miRNA in plasma after storing blood from eight healthy subjects for 4 h, 24 h, 5 days, and 7 days at RT in Streck, Roche, PAX, and Norgen tubes (**a**). Heat map representing the correlation in expression among the miRNAs tested. (**b**) qRT-PCR results showing the differences over time in miR-451a (**c**) and miR-200c (**d**) in samples stored in different tubes. Relative cf-miRNA expression compared to the 4-h time point is shown on the x-axis. Stars represent level of significance with (***) for *p*-value < 0.001.

Next, we sought to test the long-term stability of cf-miRNAs by comparing BCTs from four different companies: Streck, Roche, PAX, and Norgen. All companies claim to stabilize cfDNA for at least 7 days by using a preservative that stabilizes nucleated blood cells, thus preventing the release of nucleic acids and the contamination of RNA and DNA from healthy cells [14,15]. Norgen tubes were the only manufacturer claiming to stabilize both cfDNA and cfRNA for up to 30 days. Blood was drawn from eight healthy individuals and stored at RT for up to 7 days in each tube (Supplemental Figure S2). Total cf-miRNA content remained stable in blood stored for up to 1 week in Streck, Roche, and Norgen tubes (Figure 2a) while an elevation in cf-miRNA content was observed after 24 h in blood stored in PAX tubes (Figure 2a). On average, Roche had the least yield with 45 ng miRNA per ml plasma. Streck and Norgen were comparable with 79 and 86 ng, while PAX had the most yield with 130 ng per ml plasma, although the variability was high (Supplemental Figure S3). The hemolysis marker, miR-451a, was stable in PAX and Norgen tubes for up to 7 days (Figure 2c). In Roche tubes, miR-451a was slightly elevated after 5 days, which was significant after 7 days of storage, indicating hemolysis induction (Figure 2c). Interestingly, the strongest increase in the hemolysis marker, miR-451a, was observed in samples stored in Streck tubes for 5 and 7 days with an up to 20-fold increase compared to the initial 4-h sample. This observation correlated with the spectrophotometric analysis of heme absorption (Supplemental Figure S5). In all samples measured (*n* = 128), a strong positive correlation was observed for miRs-376c, -148b, -652, -16, and 451a (Figure 2b). The induction of hemolysis observed in the Streck and Roche tubes was reflected in the levels of the aforementioned miRNAs (Supplemental Figure S4a–d). The only miRNA which was not affected by hemolysis and remained stable in all conditions was miR-200c (Figure 2d). 

### 2.3. Circulating DNA Is Stable in Long-Term Storage Blood Collecting Tubes for up to 1 Week

**Figure 3:**
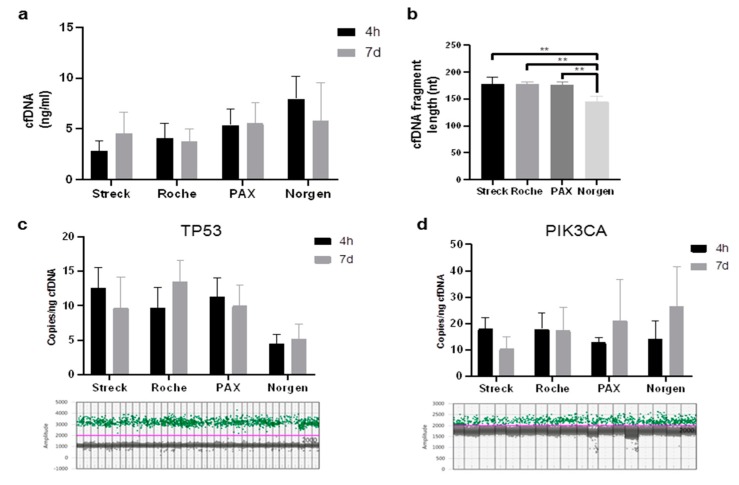
Stability of cfDNA stored in long-term storage tubes. Quantification of cfDNA in plasma after storing blood for 4 h or 7 days in Streck, Roche, PAX, and Norgen tubes (**a**). Representative DNA fragment analysis from each tube type (**b**). Quantification of *TP53* copies per ng cfDNA input; representative 1D scatterplots of *TP53* detection by droplet digital PCR (ddPCR) are depicted (**c**). Quantification of *PIK3CA* copies per ng cfDNA input; representative 1D scatterplots of *PIK3CA* detection by ddPCR are depicted (**d**). Grey: negative droplets, green: wild-type copies. Stars represent level of significance with (**) for *p*-value < 0.01.

Next, we aimed to analyze cfDNA stability in plasma from five of the eight subjects above, as future studies may want to combine cf-miRNA and cfDNA markers for disease diagnosis. We focused on the 4-h and 7-day timepoints, as all vendors claimed to stabilize cfDNA for at least 7 days. DNA content remained stable in all conditions (Figure 3a), with the most cfDNA extracted from samples stored in Norgen tubes (Supplemental Figure S6), closely followed by PAX tubes. The least amount was extracted from Streck and Roche tubes (Supplemental Figure S6). In addition to cfDNA quantification, the size of cfDNA was analyzed. All tubes yielded cfDNA with similar peak sizes ranging from 168 to 180 bp, except Norgen tubes, with a significantly lower peak size at around 150 bp at both investigated time points. (Figure 3b and Supplemental Figure S7). Finally, we utilized droplet digital PCR (ddPCR) to measure the amounts of two cancer relevant genes, *TP53* and *PIK3CA* (Figure 3c,d). For *TP53*, the least amount of counts were found in samples stored in Norgen tubes (Figure 3c). For *PIK3CA*, similar copies were obtained from all four tube types (Figure 3d).

## 3. Discussion

cf-miRNAs and cfDNA in blood have great potential to serve as non-invasive markers for disease diagnosis and/or progression. However, the pre-analytical stability of circulating nucleic acids needs to be thoroughly investigated when establishing such tests for clinical use. In the current study, we analyzed the stability of cf-miRNAs and cfDNA in liquid biopsies as a prerequisite for implementing such biopsies in regular clinical practice. 

### 3.1. Cf-miRNA Stability

To our knowledge, this is the first study that has comprehensively analyzed circulating miRNA stability in long-term blood storage tubes. While EDTA monovettes are routinely used in clinical practice, it is evident that they are not suitable for long-term blood storage [16] and reliable diagnostics. Previous studies on miRNA biomarkers have implemented stringent processing conditions, requiring processing within 2 h and blood storage at 4 °C in order to minimize effects on cf-miRNA expression [13,18]. However, this is not realistic in daily clinical practice. Here, we have shown that cf-miRNAs are stable when stored in EDTA monovettes for up to 12 or even 18 h at RT or 4 °C. Hemolysis or the lysis of other blood cells leading to the release of nucleic acids, can be a major factor affecting cf-miRNA analysis. However, we also observed little induction of hemolysis after the storage of blood at RT for up to 18 h, as quantified by miR-451a. Where we did observe variation was in individual samples and miRNAs. As well as total cf-miRNA content, both miR-148b and miR-652 were significantly elevated after 18 h in just three out of the six plasma samples stored at RT. This might indicate that particular miRNAs are less stable at RT than at 4 °C and this would reduce their predictive value in plasma samples. Further studies on a larger study group would be required to prove this. For the quantifiction of cell-free nucleic acids, we utilized the Qubit instrument to measure cf-miRNA and cfDNA content in the plasma. This relatively simple method could be implemented as a quality control before using precious liquid biopsy material in experiments. In addition, Qubit might be considered as a way to normalize miRNA expression, as there is still no consensus in the field regarding this procedure. We observed a strong inverse correlation between the Qubit and Ct values for miRs-148b and -652, indicating that they are not specific markers for disease as previously described [18], but rather a reflection of the miRNA content in blood and, therefore, also potential markers for normalization. miR-16 has been used in many studies as a reference miRNA, but it is a general marker of blood cells and correlates highly with hemolysis [16]. As we have shown, not all miRNAs are affected by hemolysis. For example, miR-200c, a well studied miRNA in cancer research [19], was not affected under any condition tested. The results of our work were limited to a panel of a few miRNAs, which did not reflect the whole miRNome. Genome wide studies should be done such as in a recent study by Keller and colleagues where they found that inter-individual variations such as age are more critical factors than pre-analytical variables in miRNA biomarker identification [20].

While EDTA tubes may be suitable for up to 18 h, in realistic clinical settings, this time-frame is still too short. Many diagnostic tests are out-sourced and samples would need to be shipped over some days. For this reason, we also explored the limits of long-term BCTs, which have already shown promising results, mainly for cfDNA [14,21,22,23]. Among the four manufacturers tested, PAX and Norgen tubes were best, showing no induction of hemolysis for up to 7 days. Roche tubes also performed well for miRNAs, with only a slight induction of hemolysis after 5 and 7 days, as well as very stable Qubit miRNA measurements. In contrast to other reports, Streck performed the worst, with a huge increase in blood cell contamination after 5 days, as assessed by miR-451a expression and heme absorption. Other studies reported satisfying results for both Streck and PAXgene tubes when using a larger cohort [15,24]. In summary, PAX and Norgen tubes performed the best at stabilizing cf-miRNAs and are, therefore, the most suitable candidate BCTs for clinical use when performing liquid biopsies.

### 3.2. cfDNA Stability

As the BCTs tested were specifically designed to preserve cfDNA and as a combination of molecular markers might be important in future diagnostics, we also analyzed the content and stability of cfDNA over time. There were no major changes in the amount of cfDNA over time. Roche tubes were most stable, both in DNA content and integrity. We found a slight increase in cfDNA content in Streck tubes, correlating with the observed hemolysis. However, none of the results were significantly different, probably due to the fact that we only included five test subjects in this experiment. With so few subjects, it is quite possible that the fluctuations can be attributed to handling or variation in the subjects. Norgen outperformed the other manufacturers in the amount of cfDNA isolated. Norgen BCTs are advantageous as they do not use formaldehyde fixation, which is a harsh treatment, and the manufacturers claim that blood can be stored for up to 30 days before processing. In addition, they are compatible for the extraction of both cfDNA and cfRNA, whereas the other manufacturers supply separate tubes for RNA analysis, making it costlier, not only financially but also in terms of the sample amount available. Therefore, for combinatorial marker studies, Norgen tubes are desirable. 

The size profiles of extracted cfDNA might give additional information about the capacity of blood stabilization. All tubes had similar peak sizes for cfDNA ranging from 168 to 180 bp, with Norgen tubes being the exception, with peak sizes around 146 bp for both time points. The length of DNA wrapped around one nucleosome is 146 bp and this might therefore recapitulate the length of cfDNA in circulation [25]. Larger fragments might indicate that cfDNA originating from blood cells was released during pre-analytical processes. The relative amounts of the two cancer-associated genes *TP53* and *PIK3CA* were comparable in cfDNA extracted from all the different tubes. As described in previous studies, highly expressed genes are underrepresented in circulation due to their low nucleosome occupancy [26]. The consistently lower copies per cfDNA detected in Norgen tubes might be indicative of this fact, while for the other tubes, blood cell-derived DNA may have contributed to the number of detected copies. In order to evaluate the diagnostic value of these indications, further investigations should be made with higher amounts of tumor-derived cfDNA. 

Unfortunately, other variables relevant to the real-work practice were not included in our study, such as the effects of sample shipment. Also, we did not subject samples to extreme temperatures higher than RT which might occur during shipping or in warmer climates. Therefore, we cannot estimate how physical forces like movements or temperature fluctuations might affect the yield and stability of circulating nucleic acids. 

The positive outcome of this work is the demonstration that plasma prepared from whole blood is suitable for the quantification of both cf-miRNAs and cfDNA simultaneously. In contrast, other studies have shown that plasma prepared for cfDNA is not suitable for miRNA analysis [23]. In this particular study, only Streck tubes were tested, which showed an induction of hemolysis over time, in concordance with our work. According to our measurements of miR-451a, no hemolysis was observed in PAX and Norgen tubes for up to 1 week, indicating that either of these tubes may be the best choice when analyzing both cf-miRNAs and cfDNA stored for up to 1 week. Roche tubes would also be sufficient for both analyses for up to 5 days. 

To conclude, the successful establishment of liquid biopsy must meet the criteria of relevant biomarkers, as well as the prerequisite of successful clinical handling, which begins with the choice of correct BCTs.

## 4. Methods

### 4.1. Blood Sample Collection and Processing

For each study, blood was drawn from healthy individuals of different ages, genders, and ethnicities (Supplemental Table S1) by standard venipuncture using a Sarstedt Safety-Multifly® 21 g canula. All participants provided consent to participate. In the first experiment, blood was drawn directly into 9-mL K3 EDTA S-monovettes ® (Sarstedt, Nümbrecht, Germany). For the second experiment, blood was drawn into long-term storage BCTs from four different manufacturers—Streck (Cell-Free DNA®, Omaha, NE, USA), Roche Diagnostics (Cell-Free DNA Collection Tube), PreAnalytiX (PAXgene Blood ccfDNA Tube, Hombrechtikon, Switzerland), and Norgen Biotek Corp. (cf-DNA/cf-RNA Preservative tubes, Thorold, ON, Canada) using the Sarstedt BloodCulture Adapter Universal. Tubes were inverted 10 times immediately after blood drawal as recommended by the manufacturers. Samples were stored upright at either RT or 4 °C for the EDTA monovettes or at RT only for BCTs before further processing at specific time points after collection. 

Plasma seperation was performed according to the laboratory’s two-step centrifugation protocol: blood tubes were centrifuged at 1300 × *g* for 20 min at RT. The plasma supernatant (one-spin plasma) was transferred into 2-mL micro centrifuge tubes followed by a second high-speed centrifugation step at 12,000 × *g* for 10 min at RT to remove cell debris and fragments. Plasma was immediately stored at −80 °C until further use. 

### 4.2. Heme Absorption

Heme absorption at 414 nm was detected from 2 µL of plasma using the Nanodrop instrument (Thermo Fisher Scientific, Waltham, MA, USA) as previously described [11]. 

### 4.3. Cf-miRNA Isolation

Cf-miRNAs were isolated from 300 µL thawed plasma using the NucleoSpin miRNA Plasma kit (Macherey-Nagel, Düren, Germany) according to the manufacturer’s procotol. Total cf-miRNAs were quantified using the Qubit microRNA Assay Kit and the Qubit Fluorometer 3.0 (Thermo Fisher Scientific, MA, USA). 

### 4.4. qRT-PCR

Two microliters of purified cf-miRNA was synthesized to cDNA using the universal Taqman Advanced miRNA cDNA synthesis kit. Individual miRNAs were amplified and quantified using Taqman Advanced specific primers and the Taqman Advanced Mastermix (Thermo Fischer Scientific) according to the manufacturer’s protocol on the qTOWER instrument (Analytical Jena, Germany). Two replicates were performed for each sample.

### 4.5. cfDNA Isolation

Plasma was thawed and cfDNA was isolated from 2 mL using the QIAmp circulating nucleic acid kit (Qiagen, Hilden, Germany) according to the manufacturer’s procotol. cfDNA was quantified using the Qubit dsDNA HS Assay kit and the Qubit Fluorometer 3.0 (Thermo Fisher Scientific). DNA size distribution was assessed on the Bioanalyzer instrument using the DNA High Sensitivity Kit (Bioanalyzer, CA, USA).

### 4.6. Droplet Digital PCR

The QX200 ddPCR system (Bio-Rad) was used with custom TaqMan probes, namely FAM and HEX, and primers (Merck KGaA, Darmstadt, Germany) designed for *TP53* and *PIK3CA* according to Bio-Rad guidelines. All reactions were prepared using the ddPCRSupermix for probes (no dUTP) (Bio-Rad, Munich, Germany), input cfDNA ranged from 0.1 to 1 ng, with 900 nmol/L of each primer, and 250 nmol/L of each probe. Each 20-μL sample was added to 70 μL of Droplet Generation Oil (Bio-Rad, Munich, Germany) and used for droplet generation (QX200 Droplet generator). All nano-emulsions underwent standard cycling conditions (10 min at 95 °C, 39 cycles of 94 °C for 30 s, 63.5 °C for 20 s, 72 °C for 30 s, followed by 98 °C for 10 min) that were optimized a priori for each primer pair using a thermal gradient for the proper annealing and reduction of cross-reactive species. After cycling, samples were transferred to a droplet reader (Bio-Rad Technologies) for fluorescent measurement of FAM and HEX probes. Droplets were scored as positive or negative based on their fluorescence intensity, which was determined by gating a threshold in no template controls. The QX200 ddPCR system consistently generated between 10,000 and 18,000 accepted droplets and the mean number of copies per droplets in the assays varied according to the input cfDNA. Three replicates were performed for each sample. The number of copies/µL was calculated using the QuantaSoftver 1.7.4 software (Bio-Rad, Munich, Germany). These values were then corrected for the input concentration to express the number of copies/ng input DNA in order to compare the different samples.

### 4.7. Statistical Analysis

Relative gene expression levels (based on the raw C_T_ values) were calculated using the 2^−∆∆*C*T^ method of Livak and Schmittgen [27]. Matrix correlations were calculated using the Statistical Tools for High-Throughput Data Analysis website [28]. Student *t*-tests were performed using Graph Pad Prism 8 software to determine significance between groups, represented by stars with (*) for *p*-value < 0.05, (**) for *p*-value < 0.01, and (***) for *p*-value < 0.001.

## Supplementary Materials

Supplementary materials can be found at http://www.mdpi.com/1422-0067/20/3/704/s1.

## Abbreviations

miRNAs	microRNAs
cfDNA	cell-free DNA
cf-miRNA	cell-free miRNA
BCTs	blood collection tubes
RT	room temperature
