# Supplementary material for: Evaluation of Storage Tubes for Combined Analysis of Circulating Nucleic Acids in Liquid Biopsies
Source: Int J Mol Sci. 2019 Feb 6;20(3):704. doi: 10.3390/ijms20030704 (PMC6387045; doi:10.3390/ijms20030704)
Supplement: Supplementary file 1 [file ijms-20-00704-s001.pdf]

Supplemental Table 1

Summary of subject characteristics from both experiments

Experiment	Subjects	Female	Male	Age range	Median age
EDTA tubes	6	3	3	23-38	29,5
Long-term storage tubes	8	5	3	23-32	25,3

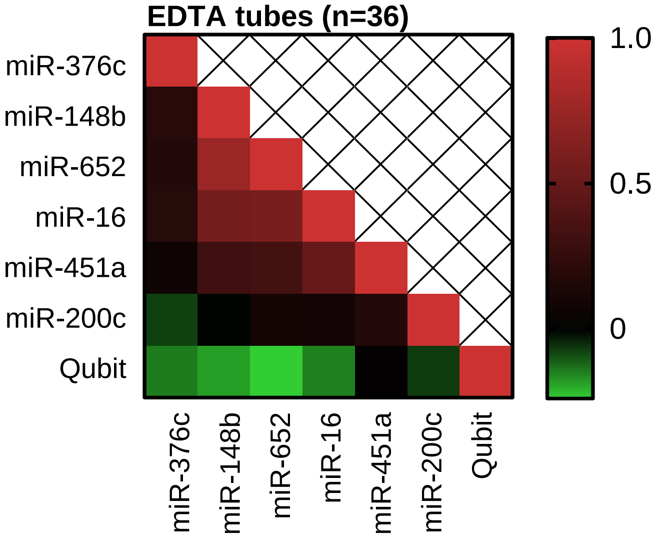

Supplemental Figure 1: Correlation of miRNAs stored in EDTA monovettes
Heatmap showing the correlation among the individual miRNAs and Qubit quantification in the experiment with EDTA tubes only.

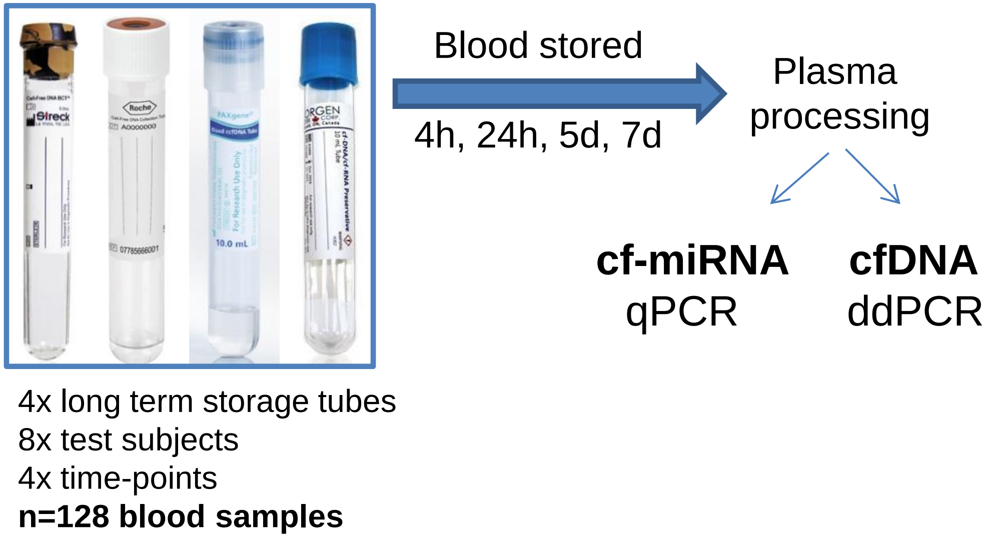

Supplemental Figure 2: Experimental Outline
Blood was drawn from 8 healthy donors into 4 different collection tubes and stored for up to 7 days before processing into plasma to be used for downstream analysis.

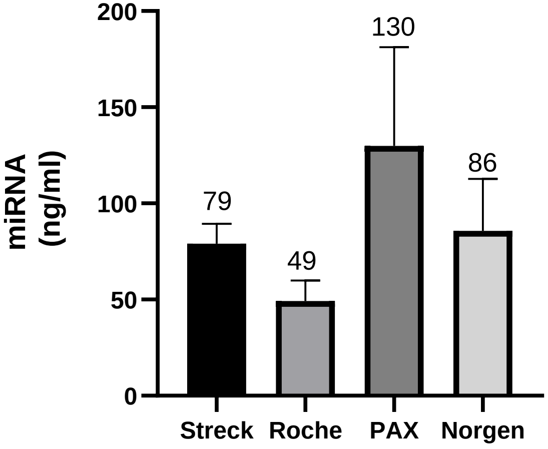

Supplemental Figure 3: miRNA content in long-term storage tubes
Average amount of cf-miRNA obtained from each tube type is shown

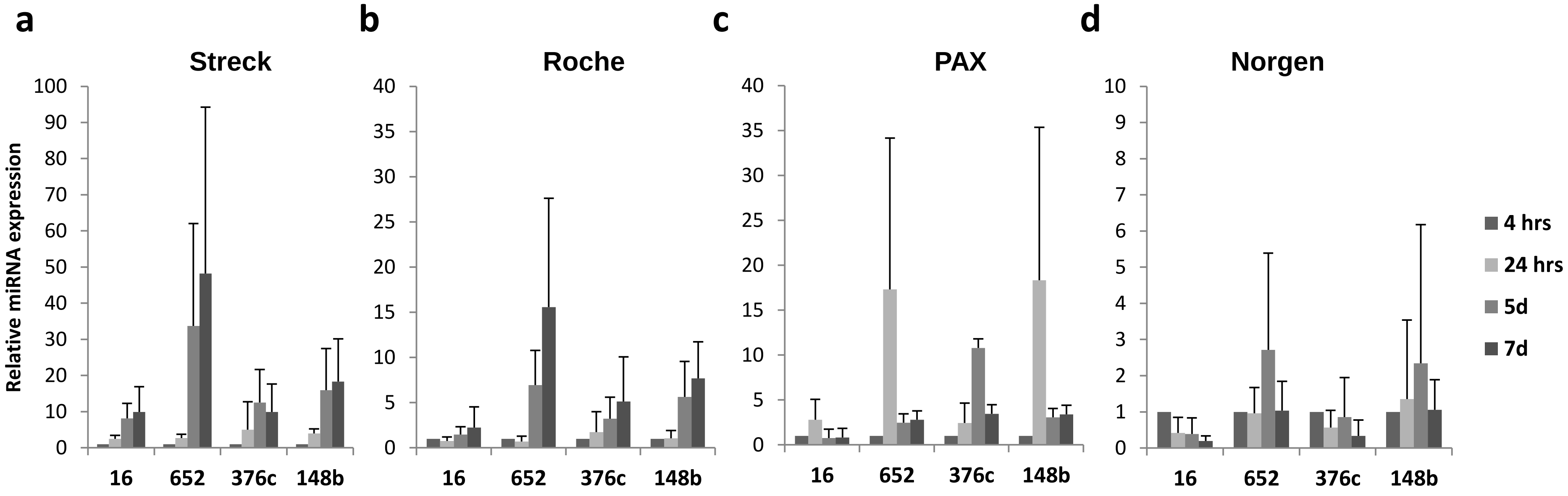

Supplemental Figure 4: miRNA qPCR
qRT-PCR for expression of miRs-16, -652, -376c and -148b in Streck (a) Roche (b) PAX (c) and Norgen (d) over time.

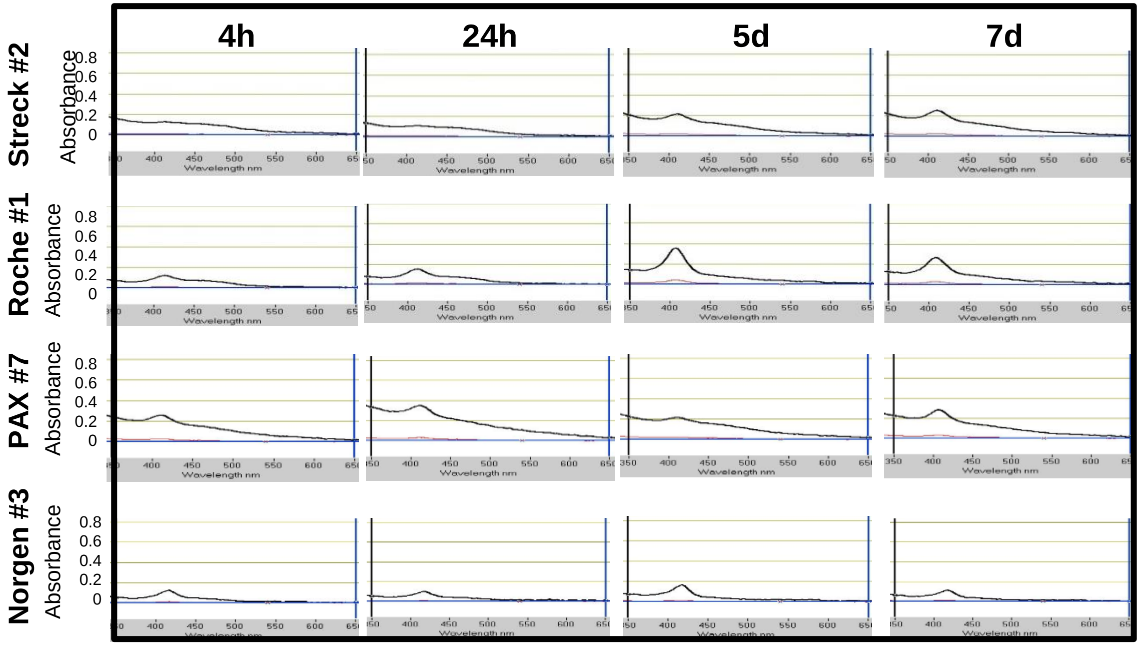

Supplemental Figure 5: Heme absorption
Representative plots for one subject from each tube type. Heme absorption, a characteristic of hemolysis, can be depicted with a peak at 414nm.

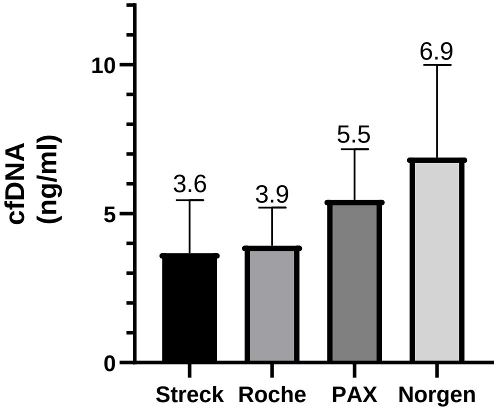

Supplemental Figure 6: cfDNA content in long-term storage tubes
Average amount of cfDNA obtained from each tube type

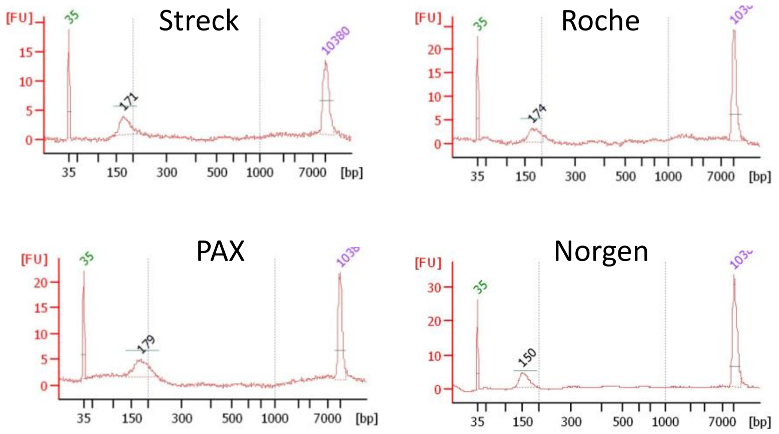

Supplemental Figure 7: Size profiles of cfDNA
A representative cfDNA fragment length from each tube type is plotted.
